# Hypoxia Inducible Factors’ Signaling in Pediatric High-Grade Gliomas: Role, Modelization and Innovative Targeted Approaches

**DOI:** 10.3390/cancers12040979

**Published:** 2020-04-15

**Authors:** Quentin Fuchs, Marina Pierrevelcin, Melissa Messe, Benoit Lhermitte, Anne-Florence Blandin, Christophe Papin, Andres Coca, Monique Dontenwill, Natacha Entz-Werlé

**Affiliations:** 1 UMR CNRS 7021, Laboratory Bioimaging and Pathologies, Tumoral Signaling and Therapeutic Targets team, Faculty of Pharmacy, 74 route du Rhin, 67405 Illkirch, France; quentin.fuchs@unistra.fr (Q.F.); marina.pierrevelcin@etu.unistra.fr (M.P.); melissa.messe@etu.unistra.fr (M.M.); benoit.lhermitte@chru-strasbourg.fr (B.L.); monique.dontenwill@unistra.fr (M.D.); 2 Pathology Department, University Hospital of Strasbourg, 1 avenue Molière, 67098 Strasbourg, France; 3 Pediatric Oncology, Dana Farber Institute, Boston, MA 02215, USA; anneflorence.blandin@gmail.com; 4 Inserm U1258, UMR CNRS 7104, Institut de Génétique et de Biologie Moléculaire et Cellulaire (IGBMC), Université de Strasbourg, 67400 Illkirch, France; papin@igbmc.fr; 5 Neurosurgery, University Hospital of Strasbourg, 1 avenue Molière, 67098 Strasbourg, France; hugoandres.coca@chru-strasbourg.fr; 6 Pediatric Onco-Hematology Department, Pediatrics, University hospital of Strasbourg, 1 avenue Molière, 67098 Strasbourg, France

**Keywords:** high-grade gliomas, pediatric, hypoxia, HIFs

## Abstract

The brain tumor microenvironment has recently become a major challenge in all pediatric cancers, but especially in brain tumors like high-grade gliomas. Hypoxia is one of the extrinsic tumor features that interacts with tumor cells, but also with the blood–brain barrier and all normal brain cells. It is the result of a dramatic proliferation and expansion of tumor cells that deprive the tissues of oxygen inflow. However, cancer cells, especially tumor stem cells, can endure extreme hypoxic conditions by rescheduling various genes’ expression involved in cell proliferation, metabolism and angiogenesis and thus, promote tumor expansion, therapeutic resistance and metabolic adaptation. This cellular adaptation implies Hypoxia-Inducible Factors (HIF), namely HIF-1α and HIF-2α. In pediatric high-grade gliomas (pHGGs), several questions remained open on hypoxia-specific role in normal brain during gliomagenesis and pHGG progression, as well how to model it in preclinical studies and how it might be counteracted with targeted therapies. Therefore, this review aims to gather various data about this key extrinsic tumor factor in pHGGs.

## 1. Introduction

Pediatric high-grade gliomas (pHGG) are aggressive and incurable tumors of the central nervous system (CNS). These infiltrative solid tumors account for 15% to 20% of all pediatric brain tumors and represent the leading cause of morbidity and death related to cancer during childhood and adolescence [1]. Only forty percent of the pHGGs originated from cerebral hemispheres and the remaining 60% are located in the midline (half in the brainstem and pons and half in the thalamus) [2]. The common standard protocol to treat hemispheric pHGG is largely inspired by adult glioblastomas and starts with surgical resection of the tumor, followed by radiotherapy and chemotherapy [3,4,5]. Unfortunately, with this type of protocol, up to 80% of children will finally relapse in the two following years, more rapidly than adult high-grade gliomas (aHGGs). In midline pHGGs, due to the widespread invasiveness of HGG cells, complete surgical resection is quite complex with the coexistence side by side of malignant and healthy cells. While many other pediatric cancers have experienced therapeutic management advances in recent decades, the median survival of pHGG has remained unchanged, with a range from 12 to 15 months in hemispheric and thalamic locations and from 9 to 12 months in Diffuse Intrinsic Pontine Gliomas (DIPGs). For all these reasons, there is a pressing need for novel therapeutic approaches to treat pHGGs. The first step was to understand and characterize intrinsic and extrinsic tumor features to reproduce pHGG conditions and be able to model it in in vitro and in vivo preclinical studies.

Since 2010 [6,7,8], clearer intrinsic pHGG features have arisen from molecular and genomic studies that led to the update in 2016 of the World Health Organization (WHO) classification of CNS tumors and its continuous improvement [9,10]. These molecular insights differentiated pHGGs from their adult counterparts, except for thalamic locations, where similar genomic features can be described in both [11]. Those studies evidenced driver mutations on histone H3 and their impact on patient outcome, linking H3.3 mutations to a specific brain location and to a worst evolution compared to H3.1 mutation [2,12,13]. Additionally, to those genomic modifications, several mutations or CNVs (Copy Number Variations) are also described mainly on *ACVR1* (Activin A receptor type I), *TP53*, *EGFR* (Epidermal Growth Factor Receptor), *ATRX* (Alpha-Thalassemia/mental Retardation X-linked)*, PTEN* (Phosphatase and TENsin homolog) or *PDGFRA* (Platelet Derived Growth Factor Receptor Alpha) [2,6,7,8,12,13,14]. Other rare driver mutations are also observed on *BRAF* or *IDH1* (Isocitrate DeHydrogenase 1). All these molecular abnormalities opened the path to new treatment approaches, but also increased knowledge on tumor heterogeneity and, thus, tumor complexity [15,16]. These molecular aspects are fundamentally different from those observed in aHGGs, explaining the specificities of pHGG and how they are divergent from the aHGGs in terms of cellular origins, epidemiology, genetic complexity, driver mutations, but also their specific extrinsic tumor features such as interactions with local hypoxic environment and brain locations [17].

**Figure 1:**
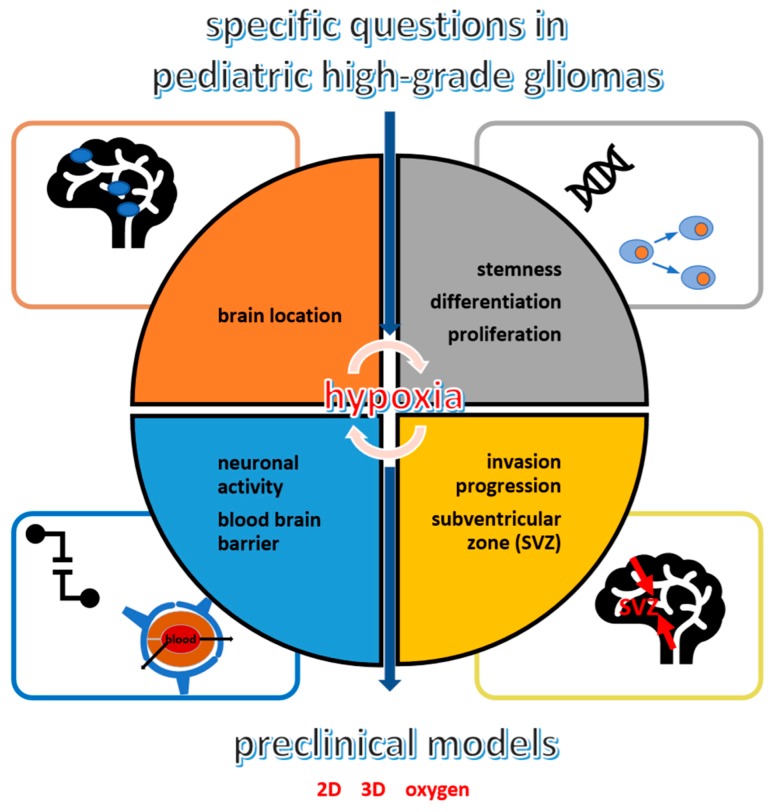
Specific questions on pediatric high-grade gliomas (pHGGs): hypoxia links with locations (orange), cell properties (grey), propensity to migrate especially from and in a subventricular zone (SVZ) (yellow) and the neuronal/blood–brain barrier environment (blue).

For extrinsic tumor features, studies mainly focus on brain microenvironment, taking into account vascular cells, brain cells and their electric activity, but also immune cells [18,19]. As most solid tumors, one key of the brain microenvironment in pHGGs seems to be hypoxia, as a result of the dramatic proliferation and expansion of tumor cells that deprive the tissues of oxygen inflow [18,20,21,22,23,24,25]. Basically, hypoxia has deleterious effects on normal cells that rapidly undergo apoptosis or necrosis after prolonged oxygen deprivation [26]. However, cancer cells, especially tumor stem cells, can endure extreme hypoxic conditions by rescheduling various genes’ expression involved in cell proliferation, metabolism and angiogenesis and thus, promote tumor expansion, resistance, and metabolic adaptation. This cellular adaptation implies HIFs (Hypoxia-Inducible Factors), namely HIF-1α, HIF-2α and HIF-3α [27,28,29]. These transcription factors mediate several key pathways in cancer and might represent interesting targets for cancer treatments, especially in pHGGs [21,24,29]. Nevertheless, several questions remained open specifically in pHGGs on the role of hypoxia in the brain during gliomagenesis, pHGG cell proliferation and widespread across normal structures, as well how to model it in preclinical studies and how it might be counteracted with targeted therapies. Therefore, this review aims to gather various data about extrinsic and intrinsic pHGG features that might interfere with intra-tumor hypoxia and where HIFs play central roles (Figure 1).

## 2. Role of Brain Physiological Hypoxia Location or Brain Physioxia and the Sub-Ventricular Zone in pHGGs

**Figure 2:**
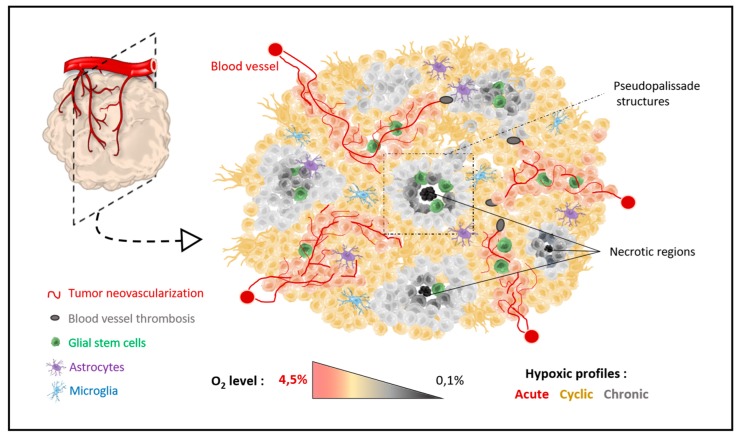
Organization of hypoxic regions across tumors. The pediatric high-grade gliomas are composed by necrotic regions surrounded by pseudopalissade structures and associated with stem cell niches. Neovascularization is starting from the periphery of the tumor and is decreasing in the center. Three hypoxic profiles are, then, described within pHGG tumors: acute, cyclic and/or chronic oxygen tensions.

Hypoxia is a general concept referring to decreased oxygen (O_2_) amounts reaching a tissue. However, in humans, physiological O_2_ levels in healthy tissues vary from 3% to 10% [21,30,31]. Thus, the physiologically hypoxic conditions are organ-dependent and can be renamed physioxia. Furthermore, O_2_ is not homogeneously distributed into one single organ but unevenly distributed in tissues, creating O_2_ gradients [32]. The brain is ones of the organs with the poorest tissue oxygen concentrations, covering a range from 3% to 4.5% [30,31,33]. This is the equivalent of an oxygen partial pressure (pO2) of about 25 to 48 mmHg, depending on altitude. Any oxygen level below this value would be considered brain hypoxia. The high brain energy requirements compared to the paradoxically low brain energy reserves implies that the brain will need continuous oxygen inflows through a very developed and active vascularization. In fact, any important oxygen fluctuation can cause nerve cell injuries. Hippocampus and cerebellum neurons seem very sensitive and hypoxia-intolerant [26], whereas neural stem-cells require hypoxic niches to remain in an undifferentiated state and maintain their pluripotency [34]. The physiological hypoxic niches are notably located in the sub-ventricular zone (SVZ) along the lateral walls of the lateral ventricles, the major site of post-natal continuous neurogenesis [23,34,35]. Controversial studies have investigated this SVZ niche as a region originating glioma stem cells [36,37]. Among those publications, signaling via mTORC1 (mammalian target of rapamycin complex 1), protein activating HIF-1α, has been proposed to regulate self-renewal, proliferative divisions, differentiation, and brain ventricle morphogenesis in this SVZ [38]. HIF-1α was also described as a repressor of BMP (Bone Morphogenetic protein) and thus, promoting HGG precursors in the SVZ niche [21]. On the other hand, glioma stem cells might directly result from the intra-tumoral hypoxic gradient [39] and explain how the glioma stem cells are located away from the SVZ [40,41,42]. They are directly initiated in the periphery of the necrotic/hypoxic regions, where HIFs are highly expressed (Figure 2).

Despite these contradictory results between SVZ and local hypoxia, it appears that physiological neural stem cells benefit from hypoxic conditions in a healthy brain and are thus capable of self-renewing to give birth to neural cells that migrate while differentiating into mature and functional neurons. Probably, these normal stem cell capacities in hypoxic niches are also used by pHGG stem cells, leading to an even more complex HGG hypoxic environment, unlimited and slow self-renewal and a high propensity to migrate rapidly [43]. This can also explain why a really frequent invasive terminal process in this SVZ is present during pHGG progression. It results from mTor/HIF-1α expression in the tumors and its close hypoxic environment [13,38,44].

## 3. Macroscopic and Microscopic Aspects of pHGG Hypoxia within the Tumors

Hypoxia in brain tumors might be closely related to the dramatic proliferation of tumor cells. Tumor expansion progressively deprives tissues from oxygen inflows by increasing their distance from blood vessels. In response to this oxygen deprivation, tumor cells promote neovascularization via VEGF (vascular endothelial growth factor) secretion. However, this tumor neovascularization is very anarchic; it results in the formation of altered and disorganized blood vessels and is unable to assure the complete oxygen delivery to tumor tissues [44]. Even VEGF and its receptor VEGFR hyperexpression have not frequently been observed in the molecular publications on pHGGs; angiogenic features and interactions with angiogenesis have been frequently discussed in preclinical studies, as well as in pHGG and DIPG trials [5,45,46,47,48]. For this specific blocking, usually, bevacizumab was used as single agent or in combination with chemo- or radiotherapy. The study of Castel et al. associated this pro-angiogenic signature to H3.1 *K27M* mutated tumors, but other studies using Magnetic Resonance Imaging (MRI) analyses on T2-weighted sequences showed a direct link between necrotic and hypoxic zones within pHGGs and more aggressive subtypes like H3.3 *K27M* mutated DIPG or those bearing *TP53* mutation [47,49]. Typical DIPG images have depicted it as arising from the brainstem and being diffusively involved in more than half of the pons. Surprisingly Hoffmann et al. found that the MRIs of short-term survivors were associated with a low necrosis percentage but a less frequent ring enhancement [50]. All those studies might explain a balance between level of hypoxic zones, neoangiogenesis and the high diffusive phenotype of the pHGG cells side to side with normal neurons and fibers, especially in pons.

The MRI imaging also showed a heterogeneous hypoxic environment in pHGG with various oxygen gradients inside one single tumor. In the same tumor, the oxygen level typically can differ in a spatio-temporal manner, creating pockets of regions with a low oxygen level surrounded by regions with a normal oxygen level that could vary over time and might be mostly localized at the tumor periphery (Figure 2). This process can be subdivided into acute, chronic and/or cyclic hypoxia [18,22,27,39,50,51,52]. Acute hypoxia acts on peripheral cells that are incompletely and intermittently oxygenated by an aberrant neovascularization. The temporal scale for acute hypoxia is generally considered from minutes to several hours. After 24 h of poor oxygen levels, the tumor cells are considered in a chronic hypoxic state and usually located in the furthest part of the tumor from the blood vessels. Lastly, the cyclic hypoxia might be considered as a kind of intermediary between acute and chronic hypoxia. The cells from the intermediate layer of the tumor endure very low oxygen levels for a few minutes to several days before being briefly re-oxygenated by immature blood vessels. These variations can explain HGG stem cell niches [29,36,53], as well as the microscopic architecture characterized by central pseudopalissades associated to neovessels and necrosis [54]. They outline the differences observed between peripheral cells of the tumor, which are oxygenated and bear less aggressive features, and inner core cells with less ring enhancement in the MRI and a very aggressive cell phenotype [37,50] (Figure 2). Usually, those extrinsic hypoxic-related features are correlated to a worst outcome and resistance to treatments (e.g., radio- and chemotherapy) [21,24,50].

## 4. Hypoxia Inducible Factors (HIFs) in pHGGs

Hypoxia-Inducible Factors are heterodimeric transcription factors composed of a regulating α subunit, which is sensitive to oxygen, and a constitutively expressed β subunit also named ARNT (Aryl hydrocarbon Receptor Nuclear Translocator). Three different homologous α subunits are commonly described, namely HIF-1, HIF-2 and HIF-3 [18,20,22,27,28,55]. For instance, knowledge is very limited regarding the HIF-3α role, but HIF-1α and HIF-2α are frequently described in hypoxia regulation. HIF-αs are a type of on/off switch for cellular adaptation to hypoxia. In the presence of oxygen, the regulating α subunit undergoes hydroxylation by the PHD (Prolyl Hydroxylase Domain) enzymes, which use oxygen and α-ketoglutarate as substrates and iron and ascorbate as cofactors. HIF-αs are, then, degraded through the proteasome pathway after being recognized and ubiquitinated in the presence of VHL (Von Hippel-Lindau) tumor suppressor. As oxygen level decreases, PHD is inhibited and the HIF-α subunit is no more degraded and accumulates in the nucleus, where it binds to the ARNT subunit. This heterodimeric complex can then fix the HRE (Hypoxia Response Element), inducing various genes’ expression mediating cell response to hypoxia. HIF-1α and HIF-2α, sharing 48% of their sequences, bind to the same HRE sequences and have many overlapping target genes. However, growing evidence suggests that HIF-1α and HIF-2α are differentially regulated in a time-dependent manner. In fact, HIF-1α is dedicated to acute hypoxia, whereas HIF-2α seems to be expressed during chronic phase of hypoxia to maintain immature cells and probably pHGG stem cell niches [27,29,35,53,54]. Their hyperexpressions are quite frequent in DIPG and pHGG cohorts. They are usually associated to mTor activation or PI3K (PhosphoInositide 3-kinase)/Akt signaling, just above the central node HIF-1α/HIF-2α [2,8,13,24,48]. Preclinical studies inducing HIF-2α knockdown reduced only in vitro glioma stem cell-mediated angiogenesis and increased glioma cell apoptosis, whereas HIF-1α knockdown resulted in reduced cell growth in both stem cells and non-stem cells [24,55].

## 5. HIF Target Genes in pHGG

As already described above, hypoxia, subsequently to HIFs induction, is associated to the expression of various proteins that will increase oxygen inflows in tissues through erythropoiesis (EPO (erythropoietin), transferrine, transferrin receptor) and aberrant neoangiogenesis in tissues (secretion of VEGF and iNOS (inducible Nitric Oxide Synthase)) [18,20,21,22,28]. A metabolic adaptation to these low oxygen concentrations is also present with the Warburg effect, inducing glucose transporters and glycolytic enzymes. pHGGs, like most other solid tumors, are highly glycolytic cancers producing large amounts of lactate as a metabolic by-product, increasing resistance to radiotherapy. All these genes have a common objective: adapt the pHGG cells to the newly encountered hypoxia conditions and maintain their proliferation and homeostasis [24,25,56,57].

**Figure 3:**
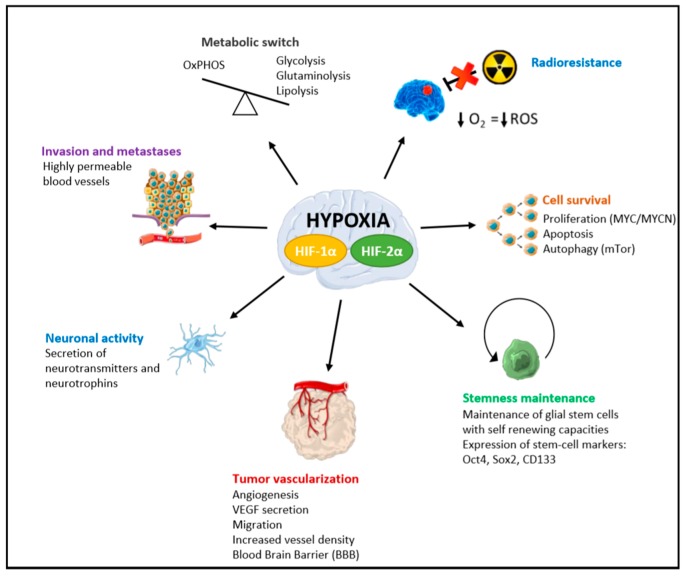
Effects of Hypoxia-Inducible Factors’ (HIF-1α and HIF-2α) hyperexpressions in pediatric high-grade gliomas. HIFs have a specific role in metabolism, radioresistance, cell survival and stemness maintenance, but also in vascularization, neuronal activity and pHGG cell migration. (OxPHOS = mitochondrial oxidative phosphorylation system, ROS = Reactive Oxygen Species).

For cell proliferation, HIF-1α and HIF-2α seem to govern differentially their downstream signaling pathways, although in opposite ways [24,27,28] (Figure 3). To regulate cell cycle progression, HIF-1α inhibits MYC, while HIF-2α promotes MYC/MYCN stabilization. On cell lines with poor MYC expression but high HIF-2α expression, this latter was sufficient to promote cell proliferation in the absence of MYC/MYCN deregulation. Indeed, the unique inhibition of HIF-2α, but not HIF-1α, was sufficient to decrease cell proliferation in MYC amplified cells, suggesting a complex transcriptional synergy between HIF-1α and HIF-2α. MYC seems also to maintain self-renewal exclusively in pHGG stem cells by selective binding to the HIF-2α promoter and the activation of the HIF-2α stemness pathway [58]. The balanced expression of HIF-1α/HIF-2α probably is one of the key drivers of pHGG cell survival and renewal concomitantly to a high level of Oct4 or CD133+ cells [40,58].

For cell homeostasis, in addition to the glycolytic switch, the mitochondrial oxidative phosphorylation system (OxPHOS) usually appears when Warburg effect decreases during tumor reoxygenation, and promotes a smaller pHGG cell viability [56] (Figure 3). However, HIF-1α and HIF-2α led the cells to a final complete metabolic reprogramming with the activation of glutaminolysis, serinolysis and/or phospholipid metabolism [24,25]. Glutamine addiction is usually associated to glycolysis impairment and Akt induction in those HGG cells [6]. This was demonstrated in patients’ tumors, as well as in pHGG-derived cell lines [24,25]. Glutaminolysis and serinolysis were associated significantly to histone *H3F3A* mutation and phospholipid metabolism to a poorer outcome [25,59,60]. These metabolic alterations represent an onco-requisite factor of pHGG progression and probably tumorigenesis. These metabolism modifications can be explored by MRI spectroscopy as well as FDG-PET (Fluoro-Deoxy-Glucose/Positron Emission Tomography) [61].

Following the metabolic modifications and linked to the oxidative stress, ROS (Reactive Oxygen Species) can increase dramatically and lead to important DNA damages, aiming to destroy tumor cells [62]. The ROS in brain tumors has long been studied since they are major criterion for radiotherapy resistance. Nevertheless, lower is the oxygen level, lower is the rate of ROS produced, resulting in lower DNA damages for the same radiation dose. Hypoxic tumors are, thus, considered as radioresistant cancers. Thus, high hypoxic regions in pHGG are predictive of poor response to radiotherapy with poor overall survival in direct correlation with ROS production. This ROS increase can also lead to autophagy induction and interplay with mTORC1 and MTORC2 hyperexpressions [38].

All those adaptive processes favor invasion and cell migration [21]. In addition, the aberrant vasculature resulting from HIF-hypoxia signaling allows a higher permeability of vessels. In addition to regular vessels, in the brain, there is a specific structure named the blood–brain barrier (BBB) that is considered as the major obstacle to adequate drug delivery in brain tumors [63,64]. The BBB is a neurovascular unit composed of endothelial cells and surrounding pericytes (Figure 1 and Figure 3). Both are characterized at the molecular level by tight junction proteins. Directly on the BBB, astrocytic feet, neurons and microglia are described. Indeed, neurogenesis and angiogenesis share regulatory factors that contribute to the simultaneous formation of vascular networks and neuronal circuits in the brain. The driving pathways of this interplay are usually based on VEGF/VEGFR balance as well as neurotransmitters like dopamine or glutamate that activate hypoxia response and the other way around [19,36,65] (Figure 3). Moreover, the interaction between hypoxic pHGG cells and neuronal activity or neurotransmitters might be now an opening field understanding further tumorigenesis and pave the way for innovative therapeutic strategies.

For instance, to our knowledge, studies in pHGGs on the interplay between immune response and hypoxia or genomic instability are very rare and would give further insight to treatment possibilities.

## 6. Comparison with Adult High-Grade Glioma (aHGG) Hypoxia

The aHGG are quite different from pHGG in various aspects, explaining the differential roles of hypoxia during their gliomagenesis. Epidemiology, clinical features, outcome, brain locations, as well as genetic and epigenetic abnormalities, are completely divergent between the two age entities.

Immunity and genomic instability are clearly two specific interplays already described with hypoxia in aHGGs. In fact, pHGGs are driven by epigenetic events, mainly mutations involving Histone 3 that are confer a hypomethylated profile, setting them clearly apart from their adult counterparts, where a predominant DNA hypermethylation is usually present [66]. These epigenetic modifications have a direct impact on tumor development and their features. DNA methylation patterns are described in adult cancers as a mechanism explaining the changes in splicing patterns under hypoxia. Recent publications in aHGG described this post-transcriptional process as a new hallmark of aggressiveness and linked intra-aHGG hypoxia to hypermethylation [67]. This RNA splicing is not present and induced in pHGG in association with hypoxia. The other additional impact of hypoxia is its role in aHGG genomic instability, where genomics, epigenomics and hypoxia are directly linked together [68].

The other main difference between adult and pediatric patients is the tumor location that we did not precisely describe in the previous paragraphs. In fact, aHGG are mainly located in hemispheres, whereas pHGG are frequently located in midline brainstem and thalamic structures. As already mentioned, O_2_ tensions and hypoxic niches are not directly located in those brain areas [21,23,34,35,43]. Nevertheless, both aHGG and pHGG invade the SVZ at the end of their evolution with differences in the invasive process under the pressure of hypoxic regions and gene deregulations [36]. The starting points in the brain of age HGG-entities may also result in differences between hypoxia locations changing during brain development and neuronal maturation during life [21,23].

All those observations bring grist to the mill for separate hypoxic-related consequences in aHGGs and pHGGs.

## 7. Hypoxia Modelization in pHGG In Vitro Studies

Today, the most accurate in vitro model for pHGG is a patient-derived cell line initiated from tumor biopsy at diagnosis or at relapse time [13,24,25,69]. Therefore, a worldwide effort was dedicated in the last decade to develop such models to mimic accurately the intrinsic patient tumor behavior. Nevertheless, in vitro hypoxia induction is underdeveloped in the preclinical pHGG studies, but as we discussed above, HIFs seem to be key drivers for pHGG cell proliferation, survival, migration and stemness maintenance. To recreate acute, chronic and cyclic hypoxic conditions in pHGG, different techniques can be proposed: either chemically hypoxia induction using Cobalt (II) Chloride hexahydrate (CoCl_2_) or Deferoxamine (DFO or DFX), either “physically” using a modular incubator chamber, 3D organoid/spheroid models or cells cultured in microenvironment matrix [24,25,32,70]. CoCl2 inhibits the PHD enzyme, which is then unable to mark HIF-1α subunit, stop its degradation and favor its accumulation in the nucleus, leading to the expression of its target genes. CoCl2 mimics only one aspect of hypoxic conditions, which is HIF-1α stabilization, but little is known about its effects on HIF-2α. We can probably obtain the same effect with recently developed PHD inhibitors [70,71]. For DFO, an iron chelator, its use in cells also leads to HIF-1α and VEGF accumulations due to MAPK (Mitogen-Activated Protein Kinase) signaling activation, with the same questioning on HIF-2α.

Finally, the only way to better reproduce the global hypoxic conditions in cells is using a hypoxic chamber or incubator in which oxygen levels can be regulated from 21% (normoxia) to the most extreme hypoxia encountered in HGG with O_2_ less than 0.1%. It can easily help us to study the variations in oxygen levels to understand the acute versus chronic versus cyclic hypoxia and then decipher the dynamics of HIF-α signaling [24,25]. Another tool that can help to quantitatively dissect the effects of hypoxia is microfluidic devices, as well as 3D and matrix environment cultures, which enable to mimic successfully the tumor microenvironment in vitro with varying concentrations of oxygen in the presence of brain cells in addition to pHGG cells and/or the BBB structures [63]. Those models are thereafter used for drug screening to find the appropriate therapies in pHGGs combined or not with irradiation, but taking into account this major microenvironment actor. In fact, the hypoxia environment impacts pHGG cell response to conventional or new therapies [24,25] through their proliferation, their metabolism and their gene expressions.

## 8. HIF Targeted Therapies

Beyond the understanding of hypoxia-related actors in pHGG, this pathway and especially HIFαs can be new therapeutic options to improve conventional chemo- and radiotherapy, but also as specific targeted therapies. Despite their close homologies in amino-acid sequence, HIF-1α and HIF-2α proteins conformations are very different and probably need specific targeting. Several mechanisms and targets can stop αα activity when inhibited: mRNA or protein HIF expression, HIF-α/HIF-β dimerization, DNA binding at HRE, transcriptional activity or increase of proteasomal degradation [71]. To target all these steps, different drugs are proposed.

For mRNA, partial or total downregulation of HIF-1α, aminoflavone, an aryl hydrocarbon receptor ligand, has been described, as well as synthetic antisense oligonucleotides, like EZN-2968. They only partially impact HIF-2α mRNA expression. The translation of HIF-α mRNA is also controlled by growth factor signaling pathways such as the PI3K/Akt/mTor pathway, that can be targeted with specific drugs. Irinotecan and topotecan are topoisomerase I inhibitors also able to downregulate HIF-1α and HIF-2 α levels [24,25].

To avoid HIF-α/HIF-β complex assembly, small molecules were recently developed to interact with PAS domains in the HIF-α and HIF-1β/ARNT subunits, like cyclic peptide inhibitor (cyclo-CLLFVY) for HIF-1α, PT2385 or PT2977 for HIF-2α and acriflavine for both HIF-αs. This blocking prevents HIFs transmission factor activity. Another way is to stop p300/CBP co-activator binding to HIF-α/HIF-β complex with chetomin, bortezomib or amphotericine B, for example. Transcriptional activation of target genes by HIFs requires a direct binding at HRE. Echinomycin or specific polyamides are those inhibitors. The last mechanism that might be used for therapeutic inhibition is an induction of HIF-1α proteasomal degradation in a VHL-independent manner (heat shock protein inhibitors) or not (histone deacetylase inhibitors). This approach is not fully understood for HIF-2α inhibition [71].

Another strategy that has been rarely, but recently, described is using drug-enhancing oxygen in the tumors, like trans sodium crocetinate [72].

In the very last trials in pHGG which have not yet been published—but information on them is available on the ClinicalTrials.gov website—the use of topoisomerase I inhibitors and PI3K/Akt/mTor targeting is frequent. As it is a frequently induced pathway in the pHGG environment, more and more studies will probably be opened or are currently recruiting with specific HIF inhibitors, like our French national RAPIRI phase I trial based on our previous preclinical work using a mTor and HIF-1α targeting [24].

Nevertheless, only partial HIF inhibition is for now available with those inhibitors and a lot of effort will be applied in the next decade to search for potent specific HIF-1α and HIF-2α antagonists. In addition to the lack of a complete antagonism of HIF-α, we might also be able to face, in the case of the entire inhibition of one single HIF-α, the balanced pathway based on HIF-1, HIF-2 and probably HIF-3 expressions and/or upstream and downstream reinduction able to overpass this inhibition. For other targeted therapies, a deep knowledge on their functional inhibition will help us to refine accurately the future trials in pHGGs.

## 9. Conclusions

In conclusion, HIF-hypoxia signaling seems to be very frequently involved in each step of pHGG development or progression and might be a new path for tumorigenesis and treatment resistance understanding. Nevertheless, we need many more pHGG studies to extend our knowledge and to select which specific groups of pHGG will benefit from these therapeutic strategies targeting the hypoxia-driven pathway. This might also be an indispensable factor to consider when developing preclinical drug screening in future pHGG trials because of its particularly high impact on cell resistance to therapies (e.g., radiotherapy or targeted drugs).
